# Vascular Transdifferentiation in the CNS: A Focus on Neural and Glioblastoma Stem-Like Cells

**DOI:** 10.1155/2016/2759403

**Published:** 2016-09-22

**Authors:** Sophie Guelfi, Hugues Duffau, Luc Bauchet, Bernard Rothhut, Jean-Philippe Hugnot

**Affiliations:** ^1^INSERM U1051, Institut des Neurosciences de Montpellier, Hôpital St Eloi, 80 Avenue Augustin Fliche, 34091 Montpellier Cedex 05, France; ^2^Université Montpellier 2, Place Eugène Bataillon, 34095 Montpellier Cedex 5, France; ^3^CHU Montpellier, Hôpital Gui de Chauliac, 80 Avenue Augustin Fliche, 34295 Montpellier, France

## Abstract

Glioblastomas are devastating and extensively vascularized brain tumors from which glioblastoma stem-like cells (GSCs) have been isolated by many groups. These cells have a high tumorigenic potential and the capacity to generate heterogeneous phenotypes. There is growing evidence to support the possibility that these cells are derived from the accumulation of mutations in adult neural stem cells (NSCs) as well as in oligodendrocyte progenitors. It was recently reported that GSCs could transdifferentiate into endothelial-like and pericyte-like cells both* in vitro* and* in vivo*, notably under the influence of Notch and TGF*β* signaling pathways. Vascular cells derived from GBM cells were also observed directly in patient samples. These results could lead to new directions for designing original therapeutic approaches against GBM neovascularization but this specific reprogramming requires further molecular investigations. Transdifferentiation of nontumoral neural stem cells into vascular cells has also been described and conversely vascular cells may generate neural stem cells. In this review, we present and discuss these recent data. As some of them appear controversial, further validation will be needed using new technical approaches such as high throughput profiling and functional analyses to avoid experimental pitfalls and misinterpretations.

## 1. Introduction

The central nervous system vasculature is singular because of the highly specialized scaffolding of the Blood Brain Barrier (BBB). Precisely, a well-organized structure called the Neurovascular Unit (NVU) participates actively in BBB integrity. It involves a close interaction between endothelial cells, mural cells, astrocyte endfeet, microglia, and neurons. Therefore, this vascular network is highly specific in its structure and components [1].

Endothelial cells (ECs) are the foundation of vessel walls. They produce the basement membrane [2], are in contact with blood flow, and closely interact with mural cells [3]. The identification of ECs relies mostly on marker expression such as CD31, CD144 (VE-Cadherin), and CD34 as well as their* in vitro* capacity to form tubular networks [4]. Considered as nonfenestrated [5], the brain endothelium is composed of three compartments that will differ in surrounding mural cells, which mainly include pericytes and vascular smooth muscle cells (vSMCs) [3, 6, 7]. The term “pericyte” was proposed by Zimmermann in 1923 to precisely define contractile cells closely surrounding microvessels but this denomination is sometimes used freely in the literature [8]. According to this definition, they must share the basement membrane with ECs [2] and physically interact with ECs at discrete membrane points [9]. Therefore, it requires a combination of histological and electron microscopy analyses, ruling out of ECs, and expression of two or more accepted “pericyte” markers to properly identify them. Contrary to those principles, many studies identify pericytes solely on the basis of markers, which are nonexclusive and overlap in expression with other perivascular cell types. These markers include PDGFR*β*, NG2, *α*SMA, desmin, and RGS5 [3, 8]. Pericytes are major regulators of vascular remodeling and tissue homeostasis. Specifically in the brain, where vascular coverage is the highest among organs, they were shown to be key players in BBB maturation and maintenance [10, 11]. Given their contractile properties, they may also regulate blood flow in response to vasoactive substances and neurotransmitters [12, 13]. Thus, ECs and pericytes are active contributors of the NVU integrity.

It has also been recently recognized that vascular cells participate in the maintenance and proliferation of neural stem cells (NSCs) within their specific neurovascular niches [14] and they can exert plasticity towards neural lineages* in vitro* [15]. In return, NSCs appear also to have vascular cell differentiation capacities [16].

Under pathological conditions, the vasculature can be acutely remodeled and expanded [6]. In this context, EC progenitors and “activated” pericytes serve as potential vascular stem cell reservoirs and intimately cooperate to ensure vascular integrity [6, 17].

Such pathological conditions include tumoral growth, where active vascularization is required to sustain malignancy of cancer cells. Glioblastoma multiforme (GBM) is highly malignant and vascularized brain tumors for which current therapeutic options are inefficient. These tumors contain subsets of radio- and chemoresistant glioblastoma stem-like cells (GSCs) that possibly originate from NSCs, thus share cardinal NSC properties, and are highly tumorigenic upon intracranial xenografts. GSCs strongly interact with vascular cells within the tumoral perivascular niche [18] and are crucial in glioblastoma-associated neovascularization mechanisms. Recently, several groups including ours have highlighted GSC plasticity towards endothelial or pericyte lineages both* in vitro* and* in vivo*.

**Figure 1:**
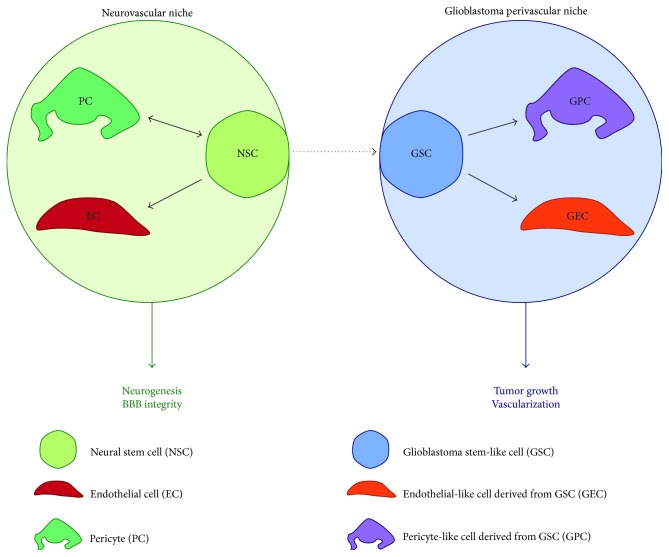
Schematic representation of the close interplay between neural stem cells (NSCs) and glioblastoma stem-like cells (GSCs) within their respective niches. NSCs are proposed to be at the origin of GSCs. Both NSCs and GSCs show transdifferentiation capacities towards the vascular lineage, that is, pericytes and endothelial cells. In both systems, this plasticity has consequences on the niche homeostasis: it influences either neurogenesis and the blood brain barrier integrity in the physiological neurovascular niche or tumoral growth and associated vascularization in the glioblastoma context.

The aim of this review is to compile recent evidences of NSC and GSC transdifferentiation towards endothelial and/or pericyte lineages, results which are actively debated in the field (Figure 1). We will discuss the intimate plasticity between vascular cells and NSCs in the physiological neurovascular niche. Then, we wish to focus on what is currently debated in the context of perivascular niches in glioblastoma.

## 2. Vascular Integrity in the CNS

Vascular wiring is one of the earliest events observed during development. The primary vascular plexus derived from the mesoderm determines arterial, venous, hemogenic, and lymphatic fates via the primordial ECs or angioblast state: a process that involves FGF2, BMP4, Indian Hedgehog (IHH), and Etv2 as molecular triggers [19–21]. The primary vascular plexus is then remodeled through a balance of VEGF signaling (VEGFR2 (Flk-1)) and TGF*β* signaling [21].

Specifically in the brain, the perineural vascular plexus surrounds the neural tube through active vasculogenesis that consequently patterns major cerebral arteries and veins. Precise vascularization within the intraneural vascular plexus is further established by angiogenic sprouting, the formation of new vessels from preexisting ones. This process includes loss of tight junction between ECs, basement membrane degradation, and migration of tip cells in association with proliferative stalk cells. Molecularly, this complex mechanism was shown to be mainly regulated by VEGF-Nrp-1, Dll4-Notch, Angiopoietins-Tie, TGF*β*, and Wnt signaling. Much less is known about angiogenic sprouting during postnatal stages. Altogether, sprouting of tip cells and anastomosis allow endothelial tube formation, later stabilized by NVU key players to form the BBB [1].

Mural cell specification is still unclear, due to speculations on a common mural precursor of mesenchymal origin for vSMCs, pericytes, and other perivascular cells [6, 7]. Most studies were performed in pathological conditions and perhaps do not reflect physiological ontogeny of mural cells. However, it is known that mural cells from coelomic structures originate from the mesothelium, whereas, in the CNS, they are in majority derived from neural crest cells [22]. Nonetheless, a common process of specification involves an epithelial-mesenchymal transition (EMT), followed by migration and colonization of mesenchymal precursors in cooperation with sprouting angiogenic ECs [8]. Signaling pathways regulating pericyte and EC crosstalk include PDGF-B/PDGFR*β*; TGF*β*; Notch1-Dll4; Angiopoietins-Tie; EphB2; and SDF1/CXCR4 signaling pathways [3, 8, 23, 24].

## 3. Adult Neural Stem Cells in the Neurovascular Niche

One of the most fascinating advances over the last three decades is represented by the discovery of immature multipotent cells in the adult brain and spinal cord of mammals. Dating back to the initial observation by Hamilton in 1901 of proliferating differentiated cells [25], the pioneer work performed by Altman and Nottebohm in the seventies [26, 27] culminated in the identification and isolation of neural stem cells (NSCs) able to produce neuronal and glial cells* in vitro* and* in vivo* [28]. The neurosphere assay [29] was instrumental in their discovery, as this assay is particularly suited to demonstrate, at the clonal level, the cardinal properties of stem cells, that is, multipotentiality, self-renewal, and extended proliferation capabilities. These multipotent cells can be divided into two classes [30]: (1)* bona fide* neural stem cells able to self-renew extensively and located in the subventricular zone and the subgranular hippocampus niches and (2) progenitors which are more proliferation/differentiation restricted cells and which are also present in the niches as well as throughout the white and gray matter. Stem and progenitor cells have also been identified in the peripheral nervous system, that is, in the carotid body, the enteric nervous system, and the adult dorsal root ganglia [31–33].

The adult brain neural stem cell niches are highly specialized structures that act as a nest and a barrier to protect, nourish, and regulate the fate of stem cells. They do so by providing cellular and molecular cues suitable for the strict control of stem cell properties (e.g., self-renewal, differentiation, and quiescence). Typically, these niches contain a high level of canonical developmental signaling pathways, notably, BMP, SHH, Wnt, and Notch. These signaling pathways precisely regulate the proliferation/quiescence, differentiation/self-renewal, and migratory/stationary balances of the stem cell pool. In addition, their particular architecture favors interactions between stem cells and specific cells, such as vascular cells, to form the so-called neurovascular niche. Actually, the state of quiescence and activation of adult stem cells is closely regulated by endothelial cells through Jagged/Dll4/Notch1, VEGFR3/VEGFC, and EphrinB2 proteins [34–36]. As regards adult brain progenitors, these cells appear to be mainly represented by oligodendrocyte precursor cells (OPCs) which could be identified by the expression of A2B5, PDGFR*α*, and NG2 [37]. These cells also called polydendrocytes, synantocytes, or NG2^+^ cells are proliferating and are involved in adult myelination.

## 4. Vascular Plasticity of Neural Stem Cells and* Vice-Versa*

The possibility that NSCs can transdifferentiate into endothelial and pericytes/smooth muscle cells was first explored in the nontumoral context. Indeed, in 2004, Wurmser et al. [16] reported that adult neural stem cell cultures cocultured with human endothelial cells expressed CD146/MCAM, a marker considered specific for endothelial cells at the time. After purification and expansion of CD146^+^ cells, Wurmser et al. then showed that these cells can express endothelial cell markers such as CD31 and CDH5, had Weibel-Palade bodies (granules found in endothelial cells), and can also generate vessel-like structures in matrix gel. They were also able to generate endothelial-like cells upon grafting in embryos. The expression of endothelial cell markers, such as CD31, as well as formation of vessels* in vitro* was observed in NSC cultures isolated from human and mouse embryos [38, 39]. Previous work also showed the remarkable capacity of NSCs to differentiate into large, flat SM-like cells that express phenotypic characteristics of SMCs [40, 41]. In addition, Oishi et al. showed that SMCs differentiated from rat CNS stem cells had the physiological characteristics of contractile smooth muscle cells [42]. However these experiments were mainly based on the expression of few markers considered to be specific for a given cell type. For instance, CD146 thought to be specific for endothelial cells in [16] is also expressed by pericytes/smooth muscle cells [43] and recent high throughput sequencing also indicated that CD146 is found in oligodendrocytes [44]. Moreover, in these studies, the authors used heterogeneous cultures derived from the whole adult brain and no or few clonal experiments were carried out. As a result, it is unclear whether these studies were based on* bona fide* NSCs derived from the VZ/SVZ or alternatively if they contained less lineage-restricted cells, possibly arising from the meninges [45] or the vessels. It remains to be confirmed whether the transdifferentiation into SMCs and endothelial cells is a general property of NSCs or if this is restricted to specific subtypes of cells.

The differentiation of neural multipotent cells into vascular cells appears not to be a one-way process as three articles reported that brain pericytes, including cells isolated from the human brain, can generate neuron-like and astrocyte-like cells by manipulating growth conditions* in vitro* [46–48]. However, this was not confirmed by two groups [49, 50]. Actually these studies mostly rely on marker expression detected by immunofluorescence, for instance, Tubb3 and Map2 for neurons and GFAP for astrocytes. However, these proteins can also be expressed by nonneural cells such as liver oval cells for GFAP quiescent hepatic stellate cells [51] and Tubb3 is a marker for lymphatic and venous valves [52]. In addition the neuronal markers Tubb3 and Map2 can also be found in glial cells [53, 54] adding further confusion to the field. As a result, it remains to be fully demonstrated that the cells which are generated are* bona fide* neurons/glial cells and not culture artefacts. It should be demonstrated that these neuronal-marker expressing cells are able to generate action potentials, make synapses, and integrate into neuronal networks. Actually, the formation of fully differentiated neurons from pericytes might require additional genetic engineering as demonstrated by Karow et al. in 2012 [15]. Indeed, this team showed that overexpression of Sox2 and Ascl1 in human and mice pericytes led to the formation of neurons able to fire action potentials and to be contacted by other neurons. This indicates that pericytes remain competent to respond to neural transcription factors to redirect their fate into neuronal cells. Specific transcriptional networks and epigenetic marks, still largely unknown, may underlie this plastic behaviour of pericytes. The multipotent properties of pericytes might also be linked to their embryonic origin. As mentioned before, brain pericytes are mainly derived from neural crest stem cells in contrast to brain neurons and glial cells which are generated from the ventricular zone. During development and* in vitro*, neural crest stem cells can generate peripheral neurons and glial cells but also smooth muscle cells. One could consider the hypothesis that brain pericytes maintain neural crest stem cell properties or even represent a pool of dormant multipotent neural crest stem cells residing in the adult brain.

## 5. Glioblastoma and Glioblastoma Stem-Like Cells

Gliomas are the most common primary brain tumors and have histologic features similar to normal glial cells, that is, astrocytes and oligodendrocytes. The precise cellular origin of gliomas remains unclear. Although traditional sources favored an origin from normal glial cells, recent data point to neural stem cells (NSCs), or NSC-derived astrocytes or oligodendrocyte precursor cells (OPCs) [55]. The current WHO classification distinguishes four grades of malignancy and glioblastoma multiforme (GBM, grade IV) is the most life-threatening, malignant, and aggressive primary neoplasm (median survival time around 15 months) which accounts for more than half of all gliomas. GBM shows histological evidence of high malignancy including nuclear atypia and high mitotic activity, along with microvascular proliferation and necrosis. Although they are histologically indistinguishable, GBM have been classified into four molecular subgroups, that is, classical, mesenchymal, proneural, and neural tumors, according to differing patterns of gene expression [56]. The classical subtype is characterized by amplification of EGFR. Although loss of the tumor suppressor PTEN and gene deletion targeting CDKN2A are frequently found, it lacks* TP53* mutation. Notch and Sonic Hedgehog pathways activation are also frequent in this subtype. The mesenchymal GBM predominantly harbors loss/mutations in the* NF1* tumor suppressor gene coding for neurofibromin 1 and shows TNF family and NFkB pathways activation and expression of mesenchymal markers such as CHI3L1, CD44, and VEGF. The proneural group harbors high oligodendrocytic marker expression (OLIG2, TCF3, and NKX2-2). It is characterized by TP53 loss/mutations, IDH1 (isocitrate dehydrogenase 1) mutations, PDGFR*α* amplification, and CpG island methylator phenotype (CIMP) expression. Most known secondary GBM, which derive from lower grade gliomas, is classified into this molecular subtype. Finally, the neural subtype typically expresses neuronal markers. Chromosome 7 copy amplification together with chromosome 10 copy loss is prevalent in the neural subtype. GBM are radio- and chemotherapy resistant and are also characterized by an abundant and abnormal vasculature. As for other cancer types, it has been recognized that GBM consist of a heterogeneous cell population, both neoplastic and nonneoplastic allowing a subset of these cells to become refractory to chemo- and radiotherapy [57–59]. In a subset of GBM, several teams including ours were able to isolate neurosphere-forming cells displaying multipotential and self-renewal properties* in vitro*. These cells express markers of OPCs and NSCs such as A2B5, Olig2, and Sox2. In orthotopic graft experiments, they have a high tumorigenic potential and are able to generate several tumoral cell types. These cells termed “glioblastoma stem-like cells” (GSCs) [60, 61] appear to be slow-growing or quiescent and reside in particular tumor niches. Recent studies also highlight the heterogeneity and dynamics of GSCs according to subtype classifications of the original tumor. Indeed, GSCs were shown to be able to transition from a proneural to a mesenchymal phenotype [62]. Moreover, another group recently proposed coexistence of multiple GSC subpopulations in tumors [63].

## 6. A Crosstalk between GSCs and Vascular Cells within the Perivascular Niche 

Extensive neovascularization is considered a major pathological hallmark of GBM. Indeed, GBM is ranked among the most vascularized solid tumors [64]. In addition to specific targeting of GSCs, targeting glioma-associated vessels represents a major therapeutic challenge.

Precisely, distinct but overlapping vascularization mechanisms have been described in the context of glioma [65]. In a brief summary, vascular cooption is considered a preliminary step towards vascularization, where invading cancer cells regroup around normal microvessels [66, 67]. Consequently, this process results in tumor growth-induced necrosis and hypoxia, which in turn trigger sprouting angiogenesis [68]. Both mechanisms employ common molecular effectors involved during physiological sprouting angiogenesis (Section 2), such as Angiopoietins/Tie2 [67, 69, 70]; VEGF [71]; Ephrins [72]; Dll4-Notch1 [73]; PDGF-B/PDGFR*β* [74]; and SDF1-CXCR4 signaling pathways [75, 76]. Angiogenesis in gliomas has also been shown to be either hypoxia dependent [70] or hypoxia independent [70, 77–79]. Vasculogenesis, which involves recruitment and differentiation of circulating bone marrow-derived cells or endothelial progenitor cells (EPCs) [80], has also been demonstrated in glioma [81] but is currently disputed and controversial, mainly because of the debate on EPC identification markers. On a molecular level, vasculogenesis share common signaling cues with angiogenesis [65]. Furthermore, vascular mimicry (VM) represents an alternative mechanism whereby vessel-like networks are formed by tumor cells at the expense of ECs. Indeed, this process has been described in human glioblastoma tissues [82, 83] and human glioma cell line xenografts [84, 85]. Altogether, these processes lead to structurally and functionally abnormal vessels, characterized by a chaotic, poorly organized vasculature with tortuous, irregularly shaped, leaky, and dysfunctional endothelial cell layer [86, 87]. In this context, endothelial cells are often loosely connected with each other and are covered by fewer and abnormal mural pericytes [87–89].

The latest mechanism of glioma neovascularization describes the capacity of glioma cells to constitute their own vasculature [65] and directly implicates the GSC subset as being able to transdifferentiate into endothelial cells (ECs) [90, 91]. We already pointed out the close interplay between NSCs and vascular cells in the physiological neurovascular niche [92]. In a comparable manner, GSCs were proposed to reside within specific tumoral perivascular niches that provide a particular microenvironment required for their maintenance and self-renewal [18, 93, 94]. This GSC niche, similarly to the NSC niche, is composed of many different key players that include not only vascular cells but also tumoral, stromal, glial, neuronal, microglial, and immune cells, as well as surrounding hypoxic and necrotic conditions [94–97].

In this context, brain ECs were shown to participate in GSC stemness through major signaling pathways that include Notch [96], Sonic Hedgehog (SHH) [98], endothelial nitric oxide eNOS [99], and HIF cascades [94]. In return, GSCs were demonstrated as active players in glioma-associated neovascularization processes [65]. Precisely, GSCs are able to secrete VEGF and SDF-1 cytokines and thus promote angiogenesis and vasculogenesis in xenograft models [100, 101]. Moreover, CD133^+^ gliospheres derived from tumors analyzed for vascular mimicry (VM) and histologically considered as VM^+^ were able to generate vascular tubule networks* in vitro*, supporting a contribution of GSCs to vascular mimicry in glioma [83]. More recently, cytokines [102] and extracellular matrix proteins [103–105] were proved to be major regulators of GSC perivascular niche integrity.

**Figure 2:**
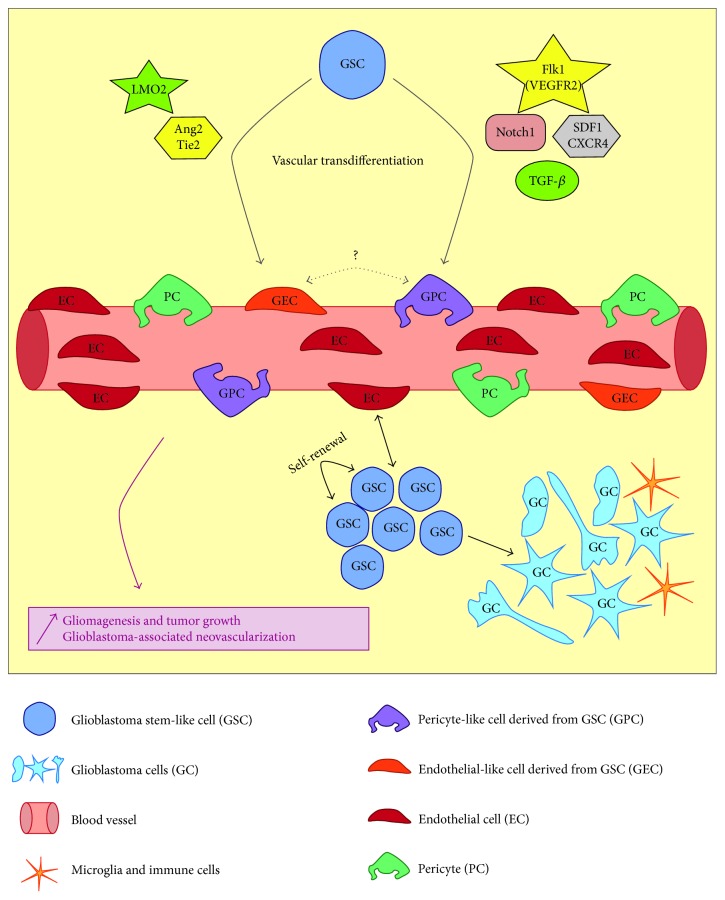
Schematic representation of the glioblastoma perivascular niche and the currently known mechanisms of GSC vascular transdifferentiation. GSCs closely interact with blood vessels in a complex perivascular niche. A close interaction with endothelial cells favors GSC self-renewal and maintenance; and in return GSCs promote neovascularization via several processes. GSCs also constitute the source of proliferating glioblastoma cells which show phenotypical heterogeneity. The tumor is also in close contact with local immune cells (microglia). Vascular transdifferentiation of GSCs into endothelial-like cells is induced via transcriptional regulation of LMO2 and also activation of Tie2 receptor. Transdifferentiation in pericyte-like cells is controlled by Notch1, TGF*β*, Flk-1, and SDF1-CXCR4 pathways. Consequently, these tightly controlled mechanisms ensure glioblastoma growth as well as tumor-associated neovascularization.

Therefore, the perivascular niche sets up unique conditions that promote both GSC survival and tumoral vascular growth (Figure 2). This aspect should be taken into account when studying the phenotypic plasticity of GSCs during transdifferentiation. Lastly, what could be considered a new mechanism of glioma neovascularization was very recently described and furthermore proved that GSCs exert peculiar plasticity. Indeed, GSCs were demonstrated as being capable of differentiating into what was defined as either mural, G-pericyte, or pericyte-like cells, complicating the picture even more. The next sections will go further into the experimental approaches and accumulating evidences for either EC transdifferentiation or pericyte-like transdifferentiation of GSCs. We will also try to point out discrepancies and what needs to be investigated in future directions.

## 7. Glioblastoma Stem-Like Cells Transdifferentiate into Endothelial Cells

First evidences of endothelial transdifferentiation of cancer cells came from melanoma and neuroblastoma studies based on clinical phenotypic analyses of patient samples [106, 107].

In the context of neuroblastoma (NB), Pezzolo et al. combined immunofluorescence of EC markers CD31 and CD105 with fluorescent* in situ* hybridization (IF-FISH) of the MYCN locus, which is commonly amplified in NB. They first observed on sections MYCN amplified ECs, in proportions that correlated with tumor grade, and ruled out pericytes as being tumor-derived. Then, they also confirmed that tumoral vasculature could be provided by cancer cells upon human NB cell line xenografts [106]. The implication of the cancer stem-like population in EC transdifferentiation was later proposed in breast cancer [108] and in a VEGF-independent fashion in ovarian cancer [109].

In GBM, GSCs were shown to be able to transdifferentiate into* bona fide* ECs in two articles that were both published in the same issue of Nature in 2010 [90, 91]. Using IF-FISH on patient sections, both studies observed ECs harboring typical GBM genetic aberrations. Both also demonstrated an* in vitro* EC differentiation capacity of GSCs isolated from freshly dissociated GBM specimens and a mainly human origin of generated vasculature upon xenografts of GSCs. In addition, Wang et al. proposed that purified CD133^+^/VE-Cadherin^−^ account for multipotent progenitors that give rise to endothelium, possibly via an EPC intermediate [90].* In vivo*, Ricci-Vitiani et al. selectively targeted ECs derived from GSCs (Tie2-tk system) and observed tumor reduction and degeneration, confirming a central function of EC transdifferentiation in tumoral progression [91]. Interestingly, some of this work is currently being reproduced as part of “The Reproducibility Project: Cancer Biology,” which “seeks to address growing concerns about reproducibility in scientific research by conducting replications of 50 papers in the field of cancer biology published between 2010 and 2012” [110].

Also, one should note that an* in vitro* study published a few months before showed an EC phenotype induction of GSCs when cultured under endothelial and hypoxic conditions. Matrigel cultures in hypoxic conditions induced tubular-like structures characteristic of typical EC networks upon electron microscopy [111]. The same* in vitro* approach was used by Dong et al. to report an EC phenotype conversion of GSCs, based on CD31, CD34, and vWF induction. In this study, xenografts experiments confirmed human origin of vessels via HLA+ staining. Moreover, in patient sample analyses, CD34^+^/nestin^+^ tumor vascular cells were found, suggesting a transitory phenotype during the transdifferentiation process [112].

Compelling* in vivo* results came from Soda et al. in 2011, who showed in a GBM mouse model that GFP^+^ tumor cells incorporated within the vasculature as tumor-derived endothelial cells (TDECs). Cell-to-cell fusion and EC progenitor contamination were ruled out to conclude that TDECs came from tumor-initiating cells. They also demonstrated that TDEC formation involved a VEGF-independent mechanism and pointed out increased treatment resistance of TDEC [113]. This correlation between EC transdifferentiation and chemoresistance was also recently shown in the context of hepatocellular carcinoma [114].

In 2013, another group found that local EPCs within patient samples could harbor glioma-associated genetic aberrations, using CD34 or VEGFR2 immunostaining combined with EGFR or PTEN FISH analyses. However, they also mentioned a blood origin of part of intratumoral EPCs, using a blood specific CD133 splice variant [115]. Also, transplantation of exogenous EPCs in a C6 glioma rat model within tumors was proposed as a potential drug delivery vehicle to target EC transdifferentiation. No impact on transdifferentiation was observed but EPC transplantation proved to be an efficient technical approach to better understand glioma vascularization [116].

Contrary to previous studies, endothelial transdifferentiation was also ruled out by others. Particularly, it was shown using fluorescent cell tracking that ECs and GSCs could form cell hybrids, pointing out potential experimental bias of previous work [117]. It was also shown in hepatocellular carcinoma that tumoral vascular networks do not arise from tumor-initiating cells, as opposed to what was proved in other works [114, 118].

On a molecular level, very few data is available concerning the transcriptional reprogramming occurring during EC transdifferentiation of GSCs. Recently, the transcription factor LMO2, which is central in hematopoietic and endothelial lineages [19, 119], was demonstrated as a potential inductor of vascular endothelial phenotype of GSCs through the direct regulation of VE-Cadherin expression. Moreover, LMO2 was shown to be expressed in GBM patient samples, but no correlation with tumoral origin of cells was made [120]. In human head and neck cancer cell lines (HNC), the Twist1-Jagged1/KLF4 transcriptional axis was proven to be essential in both EC transdifferentiation and chemoresistance [121]. In mammary tumors, the retinoic acid (RA) pathway activates a SOX9-ER81 transcriptional complex to directly induce VE-Cadherin expression and promote a vascular endothelial phenotype of cancer cell lines [122].

Whether endothelial cell transdifferentiation of GSCs can occur remains disputed and needs supplementary validation (Figure 2). In order to unravel this reprogramming, one should parallel what is currently known in physiological endothelial lineage specification to the context of tumoral transdifferentiation.

## 8. Glioblastoma Cancer Stem Cells Transdifferentiate into Mural Cells

We have already mentioned the plasticity of NSCs, that is, the capacity for these cells to differentiate into hematopoietic, muscle, and endothelial cells [16, 123, 124]. In addition, it was also shown that glioblastoma cell lines are capable of differentiating into mesenchymal lineage cell types [125] and that a subset of GSCs exhibit chondroosteogenic differentiation in response to environmental stimuli [126]. Finally since pericytes are similar to mesenchymal stem cells [127], it was worthwhile addressing the possibility that GSCs can give rise to pericytes (Figure 2).

Indeed, El Hallani et al. found in 2010 that a fraction of CD133^+^ GSCs were able to transdifferentiate into smooth muscle-like cells* in vitro* to develop vascular mimicry of the tubular type [83]. What are the cellular and molecular mechanisms underlying this pathogenesis of VM in glioblastomas? Scully et al. (2012) found that GSCs primarily transdifferentiate into vascular mural-like cells, to develop VM, a process dependent on the expression and activity of VEGF receptor 2 (Flk-1) [128]. Most recently, another important study demonstrated that GSCs are recruited towards endothelial cells by SDF-1/CXCR4 and generate pericytes mainly by TGF*β* activation. Selective elimination of GSC-derived pericytes, named G-pericytes, disrupts the neovasculature and potently inhibits tumor growth* in vivo*. G-pericytes were also identified directly in patient samples using IF-FISH combining EGFR amplification or PTEN deletion detection with *α*SMA expression [129].

Another important player in the pericyte transdifferentiation program is the Notch pathway. Our group found that overexpression of Notch1 induced a vascularization switch which was accompanied by a reduction in the growth and migration of GSCs that express several pericyte cell markers. In graft experiments, Notch1 overexpression stimulated G-pericytes association with endothelial cells [130].

Also recently, Videla Richardson et al. studied the effect of bone morphogenic protein-4 on GSC differentiation. Indeed they found that the effect was dose-dependent. At low doses, some GSC-enriched cell lines differentiated into astrocytes and neurons, whereas, at higher concentrations (10 ng/mL), they adopted a smooth muscle-like phenotype [131]. In NSCs, Rajan et al. found that at least two distinct signaling pathways are triggered by BMP4 involving the SMAD and STAT proteins. Interestingly, in cultured cells, BMP4 induced smooth muscle cell differentiation by activating SMAD1/5/8 at low basal levels of activated STAT, whereas, at higher basal levels, SMAD4 generated glia [132]. Whether these pathways are also operating in GSCs needs further investigations.

However transdifferentiation has not been confirmed by other groups. Indeed, in a recent publication, Svensson et al. using an orthotopically grafted GL261 mouse glioma model found that mice host pericytes are recruited into the tumor and that more than half of all PDGFR*β* ^+^ pericytes within the tumor are host brain-derived and do not originate from the tumor itself. However they do not rule out the possibility that the discrepancy may reside in a differential capacity of GBM plasticity and differentiation potential between different models of gliomas [133].

Another interesting crosstalk between GBM tumor cells and pericytes came from a publication by Caspani et al. providing evidence for GBM cell/pericyte fusion-hybrids formation [134], as previously described in EC transdifferentiation [117].

Does this transdifferentiation mechanism also exist in other tumors?

In infantile hemangioma (IH), it was shown that IH-derived stem cells (HemSCs) can differentiate into pericytes* in vitro* and* in vivo*, a process that is dependent on cell contact with endothelial cells [135]. In addition they show that Jagged1 is directly involved in the HemSC-to-pericyte differentiation, suggesting an important role for the Notch pathway in the formation of pathological blood vessels.

In conclusion, transdifferentiation of GSCs into pericytes is emerging as an important process in tumor angiogenesis and has to be taken into account in order to develop new therapies against glioblastoma.

## 9. Concluding Remarks and Future Directions

Here we compiled recent data supporting the transdifferentiation of normal and tumoral neural stem cells into pericytes and ECs and* vice-versa*. Whereas there is compelling evidence to support these notions, these results need to be confirmed using new techniques. In fact, most studies rely on the assessment of the expression of few genes to define pericytes, endothelial cells, neurons, astrocytes, and normal/tumoral neural stem cells. For instance, pericytes are often identified using NG2, PDGFR*β*, and *α*SMA markers which represent only a very small fraction of the genes. By measuring the expression of 3 genes among 25000 in the human genome, in fact we only assess 0.01% of all genes which could cause misinterpretations. Equally, *α*SMA is also expressed in non-smooth muscle cells such as dormant adult neural stem cells [130, 136] and also by astrocytes [137]. *α*SMA expression is also considered a molecular hallmark of epithelial-mesenchymal transition [138] and can also be induced by TGF*β* signaling in smooth muscle cells and non-smooth muscle cells [139], which could also lead to further confusion. This weak characterization of cell types produced* in vitro* and* in vivo* may lead to unreliable conclusions.

In contrast to the assessment of few markers, single cell transcriptomics analyses could be used to characterize a cell phenotype and status of differentiation in much more details. Transcriptomics profiles for a given cell type are generated through the determination not only of expression of all genes but also of noncoding RNA such as miRNA and lncRNA. As transcriptomics profiles of brain cell types are becoming available [44], this will render comparable and measurable any resemblance/or divergence in cell identity. The use of these emerging single cell techniques to explore neural stem cell *↔* pericyte transdifferentiation, in the normal or tumoral context, will certainly shed additional light on this phenomenon. This will also provide new insights into the molecular mechanisms and genes underlying this remarkable cellular plasticity. In addition to high throughput genetic profiling, electron microscopy and functional analysis would add further confirmation that endothelial cells and pericytes are obtained through transdifferentiation of GSCs.

Many questions remain unanswered regarding the transdifferentiation of GSCs into vascular cells. Does endothelial and/or pericyte transdifferentiation also occur in diffuse low grade gliomas knowing the major differences in the vasculature compared to GBM? Equally, it would be important to explore whether different molecularly defined subgroups of GBM are able to produce vascular cells or alternatively if it is restricted to GBM with particular mutations. This issue is particularly important as glioma stem cell cultures held by different labs might have different capabilities to transdifferentiate into ECs or into pericyte-like cells, which could explain the divergent results reported. One could also consider the hypothesis that GSCs could transdifferentiate through a bipotent endothelial-mesenchymal intermediate expressing both EC and smooth muscle cell markers [140], thus reconciling both models of GSCs vascular transdifferentiation.

The transdifferentiation of GBM cells into vascular cells may have implications for the appropriateness of antiangiogenic therapies as these could not only reduce the formation of vessels but also target the pool of glioma cells-derived pericytes and ECs. Finally, targeting G-pericytes could lead to a new therapeutic option to reduce tumor progression. However it has to be taken into account that only the transdifferentiated cells have to be targeted, leaving the normal pericyte population intact. Hence, it would be of great interest to find markers that could discriminate between normal and tumoral G-pericytes.
